# Berberine alleviates the cerebrovascular contractility in streptozotocin-induced diabetic rats through modulation of intracellular Ca^2+^ handling in smooth muscle cells

**DOI:** 10.1186/s12933-016-0382-9

**Published:** 2016-04-12

**Authors:** Yu-Guang Ma, Yin-Bin Zhang, Yun-Gang Bai, Zhi-Jun Dai, Liang Liang, Mei Liu, Man-Jiang Xie, Hai-Tao Guan

**Affiliations:** Department of Oncology, The Second Affiliated Hospital of Medical College, Xi’an Jiaotong University, Xi’an, 710004 Shaanxi China; Department of Aerospace Physiology, Key Laboratory of Aerospace Medicine of Ministry of Education, Fourth Military Medical University, Xi’an, 710032 Shaanxi China

**Keywords:** Berberine, Contractile function, Vascular smooth muscle cells (VSMCs), L-type Ca^2+^ channel (Ca_L_), Ca^2+^ releases

## Abstract

**Background:**

Vascular dysfunction is a distinctive phenotype in diabetes mellitus. Current treatments mostly focus on the tight glycemic control and few of these treatments have been designed to directly recover the vascular dysfunction in diabetes. As a classical natural medicine, berberine has been explored as a possible therapy for DM. In addition, it is reported that berberine has an extra-protective effect in diabetic vascular dysfunction. However, little is known whether the berberine treatment could ameliorate the smooth muscle contractility independent of a functional endothelium under hyperglycemia. Furthermore, it remains unknown whether berberine affects the arterial contractility by regulating the intracellular Ca^2+^ handling in vascular smooth cells (VSMCs) under hyperglycemia.

**Methods:**

Sprague–Dawley rats were used to establish the diabetic model with a high-fat diet plus injections of streptozotocin (STZ). Berberine (50, 100, and 200 mg/kg/day) were intragastrically administered to control and diabetic rats for 8 weeks since the injection of STZ. The intracellular Ca^2+^ handling of isolated cerebral VSMCs was investigated by recording the whole-cell L-type Ca^2+^ channel (Ca_L_) currents, assessing the protein expressions of Ca_L_ channel, and measuring the intracellular Ca^2+^ in response to caffeine. Our results showed that chronic administration of 100 mg/kg/day berberine not only reduced glucose levels, but also inhibited the augmented contractile function of cerebral artery to KCl and 5-hydroxytryptamine (5-HT) in diabetic rats. Furthermore, chronic administration of 100 mg/kg/day berberine significantly inhibited the Ca_L_ channel current densities, reduced the α_1C_-subunit expressions of Ca_L_ channel, decreased the resting intracellular Ca^2+^ ([Ca^2+^]_i_) level, and suppressed the Ca^2+^ releases from RyRs in cerebral VSMCs isolated from diabetic rats. Correspondingly, acute application of 10 μM berberine could directly inhibit the hyperglycemia-induced Ca_L_ currents and suppress the hyperglycemia-induced Ca^2+^ releases from RyRs in cerebral VSMCs isolated from normal control rats.

**Conclusions:**

Our study indicated that berberine alleviated the cerebral arterial contractility in the rat model of streptozotocin-induced diabetes via regulating the intracellular Ca^2+^ handling of smooth muscle cells.

## Background

Diabetes mellitus (DM) is a kind of metabolic diseases characterized by chronic hyperglycemia due to either reduced insulin secretion (type 1 DM) or insulin resistance (type 2 DM). DM is also considered to be a kind of vascular disorders for diabetic macro- and microvascular complications, such as coronary arterial impairment, cerebrovascular injury, and diabetic retinopathy, nephropathy, and neuropathy, are the principal causes of morbidity and mortality in patients with type 1 or type 2 DM [1, 2]. It has been demonstrated that diabetic vascular complication is associated with impaired endothelial function, augmented vasoconstriction, increased oxidative stress, enhanced inflammation, and promoted thrombosis [2]. It is believed that hyperglycemia is the hallmark feature in the development of diabetic vascular dysfunction and so current treatments mostly focus on the tight glycemic control. However, few of these treatments have been designed to directly recover the vascular dysfunction in diabetes. Because the elevated concentration of intracellular Ca^2+^ ([Ca^2+^]_i_) is the primary stimulus for smooth muscle contraction, it is reported that diabetic vascular dysfunction is tightly coupled to the impairment of intracellular Ca^2+^ handling in vascular smooth muscle cells (VSMCs) [3, 4]. Ca^2+^ influx from the long-lasting voltage-dependent Ca^2+^ (L-type, Ca_L_) channels in plasma membrane and Ca^2+^ releases from the ryanodine receptors (RyRs) in sarcoplasmic reticulum (SR) are the key factors to regulate the intracellular Ca^2+^ in VSMCs [3, 5]. An emerging body of evidence from human diabetes and different diabetic animal models indicated that hyperglycemia induced an increase of [Ca^2+^]_i_, promoted the Ca^2+^ influx by the activation of Ca_L_ channels, and altered the Ca^2+^ releases from the RyRs in VSMCs [3, 5, 6]. Therefore, the intracellular Ca^2+^ handling with their related proteins could be an important therapeutic target in diabetic vascular complications [3].

Berberine is a classical natural medicine, which has been widely used for bacteria-associated diarrhoea and other gastrointestinal infections in China [7]. In recent years, the herbal compound berberine has been explored as a possible therapy in DM for its metabolic activities of lowing blood glucose and regulating lipids, which has been studied and evidenced in the treatment of human diabetes and animal diabetic models [8, 9]. In addition, extensive research demonstrated that berberine treatment also had a cardiovascular protective effect in diabetic nephropathy, diabetic neuropathy, and diabetic cardiomyopathy [10–12]. For example, the berberine treatment was found to ameliorate diabetic endothelium-dependent relaxation by reducing oxidative stress and inflammatory response [10, 13–15]. It is believed that hyperglycemia may injury vascular function at both endothelium level and smooth muscle cell layers [2, 3]. However, previous researches on berberine mostly focused on the endothelial cells in the treatment of diabetic vascular dysfunction. Little is known whether the berberine treatment could ameliorate the smooth muscle contractility independent of a functional endothelium under hyperglycemia. Furthermore, it remains unknown whether berberine affects the smooth muscle contractility by regulating the intracellular Ca^2+^ handling in VSMCs under hyperglycemia.

The purpose of the present study was (1) to investigate the effects of berberine treatment on cerebrovascular contractile function independent of a functional endothelium in streptozotocin (STZ)-induced diabetic rats; (2) to investigate the effects of berberine on the intracellular Ca^2+^ handling of cerebral VSMCs in diabetic rats or when exposed to hyperglycemia condition, such as recording the whole-cell Ca_L_ currents, assessing the protein expressions of Ca_L_ channel, and measuring the intracellular Ca^2+^ in response to caffeine. Taken together, the present study provided initial evidences that berberine alleviates the cerebrovascular contractile function directly by the inhibition of Ca_L_ channels and the suppression of Ca^2+^ releases from the RyRs in cerebral VSMCs of streptozotocin-induced diabetic rats.

## Methods

All animal procedures described in this study were in adherence with *theGuide for the Care and Use of Laboratory Animals* published by the US National Institutes of Health (NIH Publication No. 85–23, revised 1996), with approval from Committee on the Ethics of Animal Experiments of the University of Xi’an Jiaotong University. In addition, the animal research performed in the present study was in compliance with the ARRIVE (Animals in Research: Reporting In Vivo Experiments) guidelines [16]. All surgery was performed under sodium pentobarbital anesthesia, and all efforts were made to minimize suffering unless otherwise stated, all chemicals and reagents used in this study were obtained from Sigma Chemical Company (St. Louis, Missouri, USA).

### Animal model

Male Sprague–Dawley rats (~190 g) used in this study were purchased from Medical Laboratory Animal Center of Xi’an Jiaotong University. After an adaptive feeding for 1 week, the SD rats were randomly divided into two groups: diabetic rats and control rats. The diabetic rat model was established with a high-fat diet plus injections of STZ, which was described previously [13, 17]. The high-fat diet consisted of 70 % standard laboratory chow, 5 % yolk powder, 10 % lard, 15 % carbohydrate. The control rats were given the regular diet. Following 4 weeks of dietary intervention, the diabetic rats were injected intraperitoneally with 45 mg/kg STZ for consecutively twice in 2 days, which was freshly dissolved in 0.1 M sodium citrate buffer (pH 4.5–5.0). In contrast, the control rats were injected intraperitoneally with vehicle citrate buffer in a dose volume of 1 ml/kg. Blood samples were collected by tail cutting for fasting blood glucose measurements by glucose oxidase/peroxidase method. Mean values of the fasting serum insulin level were measured by radioimmunoassay method. Successful induction of diabetes was considered as sustained fasting blood glucose levels >11.1 mM at 72 h after STZ-injection. Failed induction of diabetes was excluded during the whole study. The present study was divided into three experiments: *Experiment I, Experiment II, and Experiment III. Experiment I* was designed to investigate the effects of 100 mg/kg/day berberine on the contractile function of cerebral artery, the function of Ca_L_ channel, and the intracellular Ca^2+^ in response to caffeine in STZ-induced diabetic rats. *Experiment II and Experiment III were* designed to investigate the effects of 50 and 200 mg/kg/day berberine on the contractile function of cerebral artery, the function of Ca_L_ channel, and the intracellular Ca^2+^ in response to caffeine in STZ-induced diabetic rats. In *Experiment I*, rats were divided into four groups (n = 20/group): control rats (CON), control rats administered with berberine chloride (CON + 100 mg/kg/day berberine), diabetic rats, and diabetic rats administered with berberine chloride (Diabetic + 100 mg/kg/day berberine). In *Experiment II and Experiment III*, rats were divided into four groups (n = 10/group): control rats (CON), control rats administered with 50 or 200 mg/kg/day berberine chloride, diabetic rats, and diabetic rats administered with 50 or 200 mg/kg/day berberine chloride, respectively. After that, the diabetic and control rats were fed on the high-fat diet and the regular diet for another 8 weeks, respectively. Berberine chloride were dissolved and then intragastrically administered daily for 8 weeks. The other groups received equal volume of vehicles. The intragastric dose of berberine in this study was used according to our pre-experiments and the previous reports that the dose of 100 mg/kg/day is more close to the clinical practice than veno-injection or intraperitoneal administration [13, 18]. Eight weeks after the berberine treatment, animals were anesthetized with pentobarbital sodium (50 mg/kg ip) and killed by exsanguinations via the abdominal aorta. Body weight and fasting blood glucose were measured every week for monitoring diabetic condition. All groups were caged individually in a room maintained at 23 °C on a 12:12-h light–dark cycle and received water ad libitum.

### Examination of contractile function

As previously described [19], the segment of middle cerebral artery was transferred to the PSS containing (in mM): 119 NaCl, 4.7 KCl, 1.2 MgSO_4_, 1.2 KH_2_PO_4_, 25 NaHCO_3_, 2.5 CaCl_2_, 5.5 glucose, and 0.026 EDTA, equilibrated with 21 % O_2_, 5 % CO_2_, and 74 % N_2_ at pH 7.4 adjusted with NaOH. The endothelial layer was mechanically removed by the injection of air bubbles and then cannulated by two pipettes with nylon suture in a vessel chamber. After cannulation, the chamber was transferred to the Pressure Myograph System P110 (DMT, Denmark) and the arterial segment was perfused under a pressure of 25 mmHg for 5–10 min to check the leaking and then remove the blood residue. The arterial segment was allowed to equilibrate at 37 °C and 50 mmHg for 1 h. After equilibration, the arterial viability was evaluated by its reactivity to 20 and 60 mM isotonic KCl. Then the pressure was cycled three times between 25 and 125 mmHg to reduce mechanical hysteresis. To determine the contractile function, concentration–response relationships were determined by the cumulative superfusion of isotonic KCl (0–100 mM) and 5-hydroxytryptamine (5-HT, 10^−10^–10^−4^ M) while the arteries were pressurized at 50 mmHg in Ca^2+^-contained PSS. Contractile response to cumulative superfusion of KCl or 5-HT was represented as the percentage of luminal diameter relative to the baseline internal diameter according to the formula: luminal diameter change (%) = (Di,a,s−Di,a,b)/Di,a,b × 100 %, where Di,a,b is the baseline internal diameter measured in active state at a pressure of 50 mmHg and Di,a,s is the steady-state internal diameter measured to each subsequent change in agonist concentration at the same pressure.

### Isolation of VSMCs

Enzymatic isolation of single VSMC was carried out as previously described [20]. Briefly, brain tissues were removed rapidly and placed in 4 °C physiological salt solution (PSS) which contained (in mM) 137 NaCl, 5.6 KCl, 1 MgCl_2_, 0.42 Na_2_HPO_4_, 0.44 NaH_2_PO_4_, 4.2 NaHCO_3_, and 10 HEPES, equilibrated with 95 % O_2_ and 5 % CO_2_ at pH adjusted to 7.4 with NaOH. The cerebral arteries including superior, middle, and basilar arteries with the circle of Willis were dissected and digested for 18 min at 37 °C with the solution contained 4 mg/ml papain (Biochrom, Berlin, Germany), 2 mg/ml dithioerythritol (Amresco, St. Louis, Missouri, USA), 1 mg/ml bovien serum albumin (BSA), and 5 mM taurine in PSS. Arterial segments were then transferred to enzyme-free PSS containing 1 mg/ml BSA and 5 mM taurine at room temperature for 10 min and triturated with a flame-polished pipette to disperse VSMCs. Isolated VSMCs were suspended in Ca^2+^-free PSS containing 1 mg/ml BSA and 5 mM taurine and stored at 4 °C for use within 8 h.

### Electrophysiological measurements

As previously described [21], patch-clamp recordings were performed with an amplifier (CEZ-2300, Nihon Kohden Co., Tokyo, Japan), a version interface (Axon Instruments, Foster City, California, USA), and the pCLAMP software (version 8.0, Axon Instruments). Patch pipettes (tip resistance 2–6 MΩ) were fabricated on an electrode puller (Narishige Instruments, Tokyo, Japan). Whole-cell Ca_L_ channel currents were measured with the conventional voltage clamp configuration. Cell capacitance (Cm) was estimated from the capacitive current transient evoked by applying a 20-mV pulse for 40 ms from a holding potential of −60 to −40 mV. The cell was held at −40 mV and then stepped in 10-mV increments from −30 to +60 mV. Voltage steps were 250 ms in duration with 2-s intervals between steps. Nonspecific membrane leakage and residual capacitive currents were subtracted using the P/4 protocol. Currents were sampled and averaged while the current amplitude was stabilized. Barium (Ba^2+^) was used as the charge carrier to increase unitary currents. Currents were normalized to Cm to obtain the current densities. To obtain the *I*–*V* curve of Ca_L_, the current densities were plotted against the corresponding command potentials. Two kinds of external solutions were used, i.e., *solution A* and *B*. *Solution A* was used while making a gigaohm seal between the recording pipette and cell surface. It contained (in mM) 130 NaCl, 5.4 KCl, 1 MgCl_2_, 10 BaCl_2_, 10 HEPES, and 10 glucose, equilibrated with 95 % O_2_ and 5 % CO_2_ at pH 7.4 adjusted with NaOH. After a seal of 2 GΩ was obtained, the perfusion fluid was changed to *solution B* before current recording. It contained (in mM) 75 Tris–Cl, 50 BaCl_2_, 10 HEPES, and 10 glucose, equilibrated with 95 % O_2_ and 5 % CO_2_ at pH 7.4 titrated with Tris base. The pipette solution contained 150 CsCl, 1 MgCl_2_, 10 EGTA, 5 HEPES, 5 Na_2_ATP, and 5 Na_2_ creatine phosphate, equilibrated with 95 % O_2_ and 5 % CO_2_ at pH 7.2 titrated with CsOH. In the present study, extracellular application of 5 μM Bay K 8644 (the specific agonist of Ca_L_) and 0.1 μM nifedipine (the specific blocker of Ca_L_) were used to identify the properties of Ca_L_ as described before [21]. All measurements were performed at room temperature (22–24 °C).

### Evaluation of Ca_L_ channel protein expression by Western blotting

According to our previous report [21], cerebral arteries were minced into small pieces and homogenized on ice containing tissue protein extraction reagent (T-PER, Pierce, Rockford, Illinois, USA) and protease inhibitor (Halt, Pierce, Rockford, Illinois, USA). Large tissue debris and nuclear fragments were removed by two centrifuge spins (1000 rpm for 5 min, 12,000 rpm for 15 min) at 4 °C and supernatants were obtained. The concentration of protein sample was determined by the bicinchoninic acid method (Pierce, Rockford, Illinois, USA) using BSA as a standard. Equivalent amounts of proteins from different groups were loaded to adjacent lanes for SDS-PAGE. Protein samples were run for 80 min at 30 mA for an electrophoretic size-separation using a 8 % Tris–Glycine gel (Invitrogen, Carlsbad, California, USA). After size separation, proteins were transferred onto a nitrocellulose membrane at 100 mA for 3 h and blocked with 5 % nonfat dry milk in PBS containing 0.1 % (w/v) Tween 20 (PBS-T) overnight at 4 °C. Subsequently, the membranes were incubated for 3 h with a 1:200 dilution of rabbit polyclonal antibody against amino acids 848–865, which corresponds to the C-terminus site of Ca_L_ channel α_1C_-subunit (Alomone Labs, Jerusalem, Israel). The membrane then incubated for 45 min with Infrared (IR)-labeled secondary antibodies (LI-COR) in PBS-T containing 0.01 % SDS. A monoclonal mouse antibody raised against the structural protein β-actin (Sigma) was used as a lane-loading control. The bound antibody was detected by the Odyssey infrared imaging system (LI-COR), and the densities of immunoreactive band were expressed as a percentage of the β-actin density for each lane. Densitometry analysis of bands was performed by Scion image (Scion, Frederick, MD).

### Measurement of intracellular Ca^2+^ in response to caffeine

As previously described [21], we investigated the average changes of intracellular Ca^2+^ fluorescence intensity with Ca^2+^ indicator, Fluo-3-acetoxymethyl ester (Fluo-3/AM, Molecular Probes, Oregon, USA). Isolated cerebral VSMCs were incubated with Fluo-3/AM in a final concentration of 5 μM for 30 min at 37 °C. Then, the Fluo-3/AM-loaded cells were washed with Ca^2+^-free balanced salt solution (BSS) which contained (mM) 126 NaCl, 5 KCl, 0.3 NaH_2_PO_4_, 10 HEPES, 1 MgCl_2_, 10 glucose, 1 EGTA, equilibrated with 95 % O_2_ and 5 % CO_2_ at pH 7.4 adjusted with NaOH. The cells were scanned under a laser confocal microscope (Olympus FV1000, Tokyo, Japan) by illuminating with a krypton/argon laser at 488 nm emitted light and capturing the emitting fluorescence at 526 nm. To ensure efficient quantum capture, the cells were placed on the bottom of a recording chamber and images were recorded after 10–20 s when fluorescence intensity became stable. During continuously scanning, 10 mM caffeine in Ca^2+^-free BSS was administrated to the cell and a period of 3 min was recorded. To avoid any laser-induced change in Ca^2+^ signaling, each cell was scanned only once. The average fluorescence intensity was used to indicate the changes of intracellular Ca^2+^. The maximal increase of Ca^2+^ fluorescence intensity was used to indicate the function of ryanodine-sensitive Ca^2+^ releases from SR in VSMCs.

### Statistical analysis

Except the data of body weight are given as mean ± SD, all other data are expressed as mean ± SEM. One-way ANOVA was used to determine the differences of Ca_L_ channel current densities in different groups, followed by a S–N–K-*Post Hoc*. Student’s *t* test was used to determine significant differences of body weights, blood glucose, the resting Ca^2+^ fluorescence intensity, and the maximal increase of Ca^2+^ fluorescence intensity in different groups. A value of *P* ≤ 0.05 was considered to be statistically significant.

## Results

### Physical characteristics of experimental animals

The rat model of STZ-induced diabetes is well-established using a toxin to destroy the pancreatic β-cells. As compared with control rats, the levels of blood glucose significantly increased and the body weights significantly decreased in diabetic rats from 4 to 8 week after STZ-injection, respectively, which indicated that diabetic rat model has been established successfully [13, 18]. In *Experiment I*, when the diabetic rats were treated with 100 mg/kg/day berberine for 4 or 8 weeks, there were a marked decrease in the blood glucose and an obvious increase in the body weight of diabetic rats, respectively, which is well in accordance with earlier studies [13, 18]. However, there were also the significant differences in the blood glucose and body weight between Diabetic + berberine and CON rats, which indicated that berberine treatment did not restore the blood glucose and body weight of diabetic rats to the normal control level (Table 1). When the control rats were treated with 100 mg/kg/day berberine, there was only a declined tendency and no significant difference both in the blood glucose and body weight between CON + berberine and CON rats. In addition, as shown in Table 2, diabetic rats showed a drastic increase in serum insulin level as compared with the normal control group, which is similar to previous report [13]. After 8 weeks of treatment of 100 mg/kg/day berberine, serum insulin levels also significantly decreased in diabetic rats. These results indicated that 100 mg/kg/day berberine treatment obviously has a hypoglycemic effect in diabetic rats.

**Table 1:** Body weight and fasting blood glucose in CON, CON +100 mg/kg/day berberine, diabetic, and diabetic +100 mg/kg/day berberine rats at 4 and 8 weeks after STZ-injection in *Experiment I*

	Initial		4 Week after STZ-injection		8 Week after STZ-injection	
	Body weight (g)	Blood glucose (mM)	Body weight (g)	Blood glucose (mM)	Body weight (g)	Blood glucose (mM)
CON (n = 20)	195.0 ± 25.7	4.2 ± 1.2	423.5 ± 55.8	5.2 ± 0.9	501.0 ± 75.6	5.6 ± 1.5
CON + berberine (n = 20)	186.5 ± 28.1	3.8 ± 2.2	417.9 ± 65.3	4.1 ± 1.2	483.5 ± 72.1	4.5 ± 1.7
Diabetic (n = 20)	190.0 ± 23.4	3.6 ± 1.3	270.3 ± 46.3*	21.9 ± 5.4*	251.0 ± 52.7*	25.91 ± 6.6*
Diabetic + berberine (n = 20)	187.0 ± 25.5	4.1 ± 1.6	327.0 ± 51.2^#,^*	16.5 ± 4.5^#,^*	353.0 ± 58.3^#,^*	14.8 ± 4.3^#,^*

*CON* control rats, *CON* *+* *berberine* control rats administrated with berberine (100 mg/kg/day), *Diabetic* diabetic rats, *diabetic* *+* *berberine* diabetic rats administrated with berberine (100 mg/kg/day). * *P* < 0.05 vs. CON and^ #^ *P* < 0.05 vs. diabetic rats

**Table 2:** Mean values of the serum insulin level in CON, CON +100 mg/kg/day berberine, diabetic, and diabetic +100 mg/kg/day berberine rats at 8 weeks after STZ-injection in *Experiment I*

Group	Insulin (μIU/ml)
CON (n = 20)	9.0 ± 1.5
CON + berberine (n = 20)	8.7 ± 1.7
Diabetic (n = 20)	13.6 ± 1.8*
Diabetic + berberine (n = 20)	11.5 ± 1.2^#,^*

*CON* control rats, *CON* *+* *berberine* control rats administrated with berberine (100 mg/kg/day), *Diabetic* diabetic rats, *diabetic* *+* *berberine* diabetic rats administrated with berberine (100 mg/kg/day). * *P* < *0.05* vs. CON and ^ #^ *P* < *0.05* vs. diabetic rats

In *Experiment II* and *Experiment III,* when the diabetic rats were treated with 50 mg/kg/day berberine for 8 weeks, there were no obvious effects on blood glucose and body weight in CON and diabetic rats, respectively. However, 200 mg/kg/day berberine for 8 weeks significantly decreased blood glucose in both CON and diabetic rats (Table 3).

**Table 3:** Body weight and fasting blood glucose in CON, CON +50 or 200 mg/kg/day berberine, diabetic, and diabetic +50 or 200 mg/kg/day berberine rats at 8 weeks after STZ-injection in *Experiment II and Experiment III*

	Initial		8 Week after STZ-injection	
	Body weight (g)	Blood glucose (mM)	Body weight (g)	Blood glucose (mM)
*Experiment II*				
CON (n = 10)	187.0 ± 22.8	4.9 ± 1.8	493.0 ± 55.2	5.2 ± 2.3
CON +50 mg/kg/day berberine (n = 10)	190.5 ± 18.3	4.3 ± 2.0	511.5 ± 62.1	5.7 ± 2.1
Diabetic (n = 10)	189.0 ± 17.6	4.6 ± 1.9	253.0 ± 72.7*	19.9 ± 4.9*
Diabetic +50 mg/kg/day berberine (n = 10)	191.0 ± 18.5	4.9 ± 2.5	261.0 ± 68.7^#,^*	18.2 ± 5.3*
*Experiment III*				
CON (n = 10)	195.0 ± 23.1	4.5 ± 1.2	503.0 ± 47.9	5.6 ± 1.2
CON +200 mg/kg/day berberine (n = 10)	196.5 ± 18.6	3.9 ± 2.5	406.5 ± 57.2*	3.7 ± 1.1*
Diabetic (n = 10)	200.0 ± 21.7	4.3 ± 1.8	296.0 ± 82.1*	18.8 ± 4.5*
Diabetic +200 mg/kg/day berberine (n = 10)	192.0 ± 17.8	4.4 ± 1.6	381.0 ± 55.8^#^	10.2 ± 3.7^#,*^

*CON* control rats, *CON* *+* *berberine* control rats administrated with berberine, *Diabetic* diabetic rats, *diabetic* *+* *berberine* diabetic rats administrated with berberine. * *P* < 0.05 vs. CON and ^ #^ *P* < 0.05 vs. diabetic rats

### Chronic treatment of 100 mg/kg/day berberine significantly inhibited the augmented contractile function of middle cerebral artery in diabetic rats

**Fig. 1:**
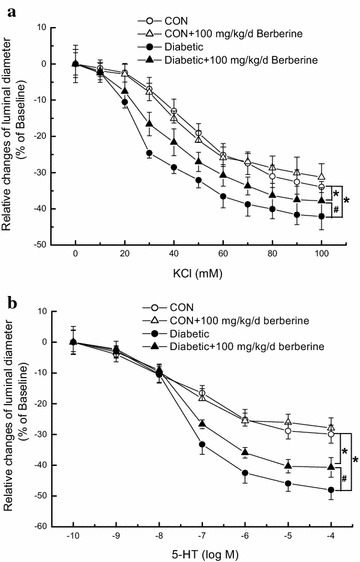
Comparison of contractile response in middle cerebral artery to cumulative superfusion of KCl (**a**) and 5-hydroxytryptamine (5-HT, **b**) in CON, CON +100 mg/kg/day berberine, diabetic, and diabetic +100 mg/kg/day berberine rats. Concentration–response relationships of middle cerebral artery were represented as the percentage of luminal diameter relative to the baseline internal diameter. Chronic administration of 100 mg/kg/day berberine significantly inhibited the augmented contractile responsiveness to KCl (**a**) and 5-HT (**b**) in diabetic rats, respectively. *CON* control rats, *CON* *+* *berberine* control rats administrated with berberine (100 mg/kg/day), *Diabetic* diabetic rats, diabetic + berberine: diabetic rats administrated with berberine (100 mg/kg/day). Values are expressed as mean ± SEM and n = 8 animals in each group. *P < 0.05 vs. CON rats and^ #^ *P* < 0.05 vs. diabetic rats

**Fig. 2:**
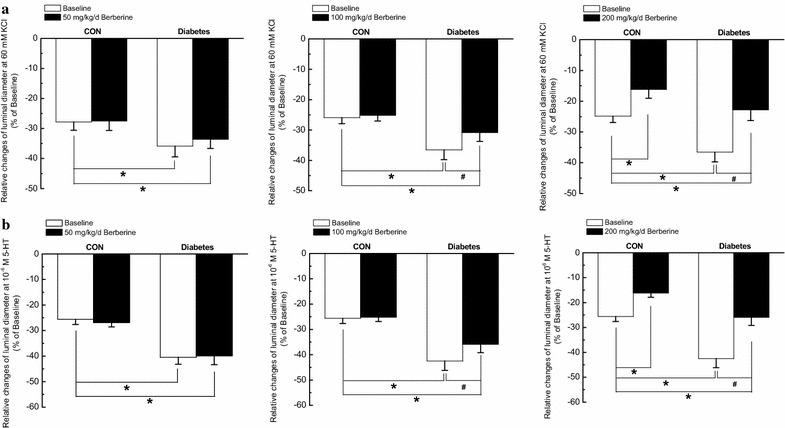
Comparison of contractile function in response to different dose of berberine (50, 100 and 200 mg/kg/day) at 60 mM KCl (**a**) and 10^−6^ M 5-hydroxytryptamine (5-HT, **b**) in middle cerebral artery isolated from CON, CON + berberine, Diabetic, and diabetic + berberine rats, respectively. Chronic administration of 50 mg/kg/day berberine had no obvious effects on contractile responsiveness to KCl (**a**) and 5-HT (**b**) in CON and diabetic rats, respectively. However, chronic administration of 100 mg/kg/day berberine did not change the contractile responsiveness in CON rats, whereas significantly inhibited the augmented contractile responsiveness to KCl (**a**) and 5-HT (**b**) in diabetic rats, respectively. In addition, chronic administration of 200 mg/kg/day berberine significantly inhibited the contractile responsiveness to KCl (**a**) and 5-HT (**b**) in both CON and diabetic rats, respectively. *CON* control rats, *CON* *+* *berberine* control rats administrated with different dose of berberine, *Diabetic* diabetic rats, diabetic + berberine: diabetic rats administrated with different dose of berberine. Values are expressed as mean ± SEM and n = 8 animals in each group. *P < 0.05 vs. CON rats and^ #^ *P* < 0.05 vs. diabetic rats

In the present study, removal of the endothelial layer was used to rule out the endothelium-dependent relaxation, which may affect the contractile responses of VSMCs. As compared with that in CON, contractile responsiveness of middle cerebral artery to KCl (Fig. 1a) and 5-HT (Fig. 1b) were both significantly increased in diabetic rats, which is in accordance with previous report [22]. In *Experiment I*, chronic administration of berberine (100 mg/kg/day) for 8 weeks significantly inhibited the augmented contractile responsiveness of middle cerebral artery to KCl (Fig. 1a) and 5-HT (Fig. 1b) in diabetic rats, respectively. For example, when the concentration of KCl was 60 mM, 100 mg/kg/day berberine treatment could markedly reduce the relative changes of luminal diameter from (−36.5 ± 3.2) % in diabetic rats to (−30.8 ± 3.0) % in diabetic + berberine rats (Fig. 2a). In addition, when the concentration of 5-HT was 10^−6^ M, 100 mg/kg/day berberine treatment could significantly decrease the relative changes of luminal diameter from (−42.5 ± 3.7) % in diabetic rats to (−35.9 ± 3.3) % in diabetic + berberine rats (Fig. 2b). However, there was also a significant difference in the contractile responsiveness of middle cerebral artery between Diabetic + berberine and CON rats, which indicated that berberine treatment did not restore the contractile responsiveness of middle cerebral artery in diabetic rats to the normal control level. When the control rats were treated with berberine (100 mg/kg/day), there was no significant difference in the contractile responsiveness of middle cerebral artery between CON + berberine and CON rats. These results clearly suggested that 100 mg/kg/day berberine treatment significantly inhibited the augmented contractile function of middle cerebral artery in diabetic rats.

In *Experiment II* and *Experiment III,* chronic treatment with 50 mg/kg/day berberine for 8 weeks had no obvious effects on contractile responsiveness in CON and Diabetic rats, respectively. However, 200 mg/kg/day berberine for 8 weeks significantly inhibited contractile responsiveness in both CON and diabetic rats (Fig. 2).

### Chronic administration of 100 mg/kg/day berberine markedly inhibited the increased Ca_L_ current densities of cerebral VSMCs in diabetic rats

**Fig. 3:**
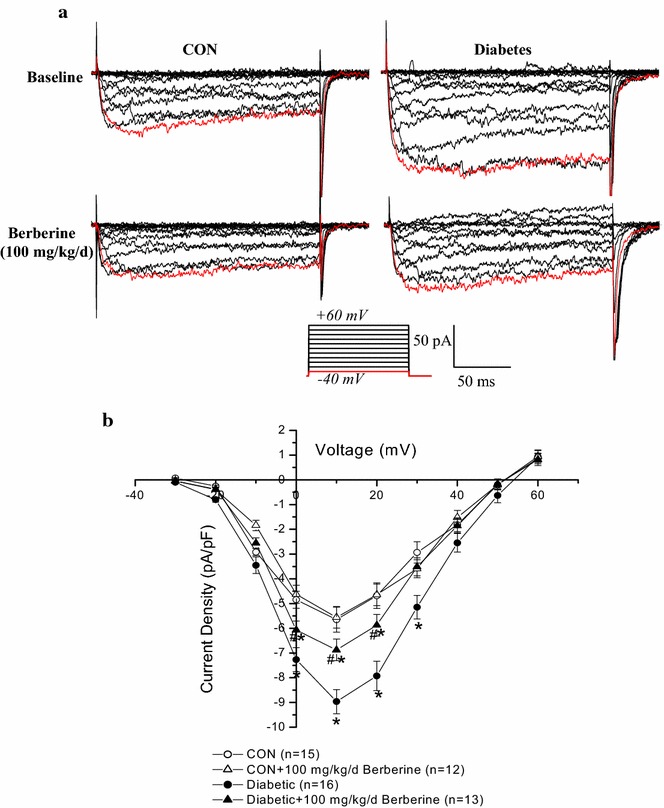
Comparison of whole-cell Ca_L_ current density in cerebral VSMCs isolated from CON, CON +100 mg/kg/day berberine, Diabetic, and diabetic +100 mg/kg/day berberine rats. Representative recording traces were used to show the whole-cell Ca_L_ currents (**a**) and the mean *I*–*V* curves were further expressed in terms of current densities (**b**) in different groups. Chronic administration of 100 mg/kg/day berberine markedly inhibited the increased Ca_L_ channel current density of cerebral VSMCs in diabetic rats. *CON* control rats, *CON* *+* *berberine* control rats administrated with berberine (100 mg/kg/day), *Diabetic* diabetic rats, diabetic + berberine: diabetic rats administrated with berberine (100 mg/kg/day). Values are mean ± SEM with the number of cells recorded in parentheses. *P < 0.05 vs. CON rats and^ #^ *P* < 0.05 vs. diabetic rats

To evaluate the function of Ca_L_ channels, whole-cell currents were recording with conventional patch clamp techniques. As shown in Fig. 3a, the typical time- and voltage-dependent inward currents were evoked by increasing depolarizations from a holding potential of −40 mV to +60 mV. The mean current–voltage relationship (*I*–*V)* curves which were further expressed in terms of current densities which were calculated by normalizing current to Cm (Fig. 3b) [20]. Whole-cell Ca_L_ currents of cerebral VSMCs in diabetic rats showed a larger inward components of trace as compared with that in CON (Fig. 3a), which is consistent with the previous reports [5, 22]. In *Experiment I*, chronic administration of berberine (100 mg/kg/day) for 8 weeks significantly inhibited the increased Ca_L_ channel current densities of cerebral VSMCs in diabetic rats. However, there was also a significant difference in Ca_L_ channel current densities of cerebral VSMCs between Diabetic + berberine and CON rats, which indicated that berberine treatment did not restore the Ca_L_ channel current densities of cerebral VSMCs to the normal control level. When the control rats were treated with berberine (100 mg/kg/day), there was no significant difference in the Ca_L_ channel current densities of cerebral VSMCs between CON + berberine and CON rats. These results clearly suggested that 100 mg/kg/day berberine treatment significantly inhibited the increased Ca_L_ channel current densities of cerebral VSMCs isolated from diabetic rats.

**Fig. 4:**
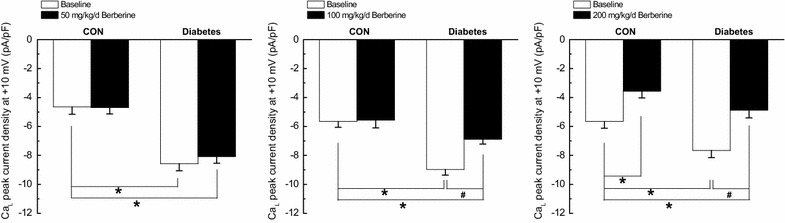
Comparison of Ca_L_ peak current density in response to different dose of berberine (50, 100 and 200 mg/kg/day) in cerebral VSMCs isolated from CON, CON + berberine, diabetic, and diabetic + berberine rats. Chronic administration of 50 mg/kg/day berberine had no obvious effects on Ca_L_ peak current density in CON and diabetic rats, respectively. However, chronic administration of 100 mg/kg/day berberine did not change the Ca_L_ peak current density in CON rats, whereas significantly inhibited the augmented Ca_L_ peak current density in diabetic rats, respectively. In addition, chronic administration of 200 mg/kg/day berberine significantly inhibited the Ca_L_ peak current density in both CON and diabetic rats, respectively. *CON* control rats, *CON* *+* *berberine* control rats administrated with berberine, *diabetic* diabetic rats, *diabetic* *+* *berberine* diabetic rats administrated with berberine. Values are expressed as mean ± SEM and at least n = 10 cells recorded in each group. *P < 0.05 vs. CON rats and^ #^ *P* < 0.05 vs. diabetic rats

In *Experiment II* and *Experiment III,* chronic treatment with 50 mg/kg/day berberine for 8 weeks had no obvious effects on Ca_L_ channel function in CON and diabetic rats, respectively. However, 200 mg/kg/day berberine for 8 weeks significantly inhibited Ca_L_ channel function in both CON and diabetic rats (Fig. 4).

### Chronic administration of 100 mg/kg/day berberine significantly reduced the augmented α_1C_-subunit expressions of Ca_L_ channel in cerebral arteries isolated from diabetic rats

**Fig. 5:**
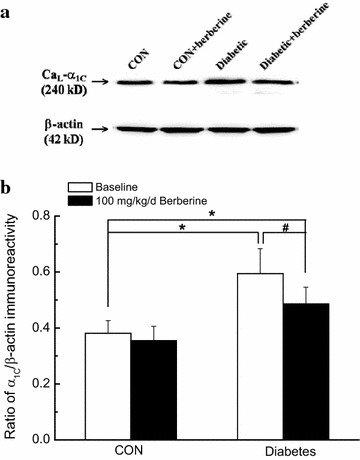
Comparison of α_1C_-subunit expressions of Ca_L_ channel in cerebral arteries isolated from CON, CON +100 mg/kg/day berberine, diabetic, and diabetic +100 mg/kg/day berberine rats. Representative band was used to show the protein expressions of Ca_L_ channel in different groups (**a**). Normalized band intensities are shown as a percentage of the β-actin density (**b**). Chronic administration of 100 mg/kg/day berberine markedly reduced the augmented α_1C_-subunit expressions of Ca_L_ channel in cerebral arteries isolated from diabetic rats. *CON* control rats, *CON* *+* *berberine* control rats administrated with berberine (100 mg/kg/day), *Diabetic* diabetic rats, *Diabetic* *+* *berberine* diabetic rats administrated with berberine (100 mg/kg/day). Values are expressed as mean ± SEM from four independent experiments, and each sample based on tissue pooled from 3–4 animals. *P < 0.05 vs. CON rats and^ #^ *P* < 0.05 vs. diabetic rats

The apparent masses of 240 kD band were shown in the membrane, which correspond to the predicted sizes of α_1C_-subunit protein of Ca_L_ channel in cerebral arteries [21]. As the internal control, the expressions of β-actin (42 kDa) were similar in different lanes in the bottom membrane, showing the equal protein loading (Fig. 5a). The averaged data was expressed as the percentage of β-actin signal (Fig. 5b). As compared with that in CON rats, α_1C_-subunit expressions of Ca_L_ channel significantly increased in cerebral arteries of diabetic rats. In *Experiment I*, chronic administration of berberine (100 mg/kg/day) for 8 weeks significantly reduced the augmented α_1C_-subunit expressions of Ca_L_ channel in cerebral arteries of diabetic rats. However, there was also a significant difference in the α_1C_-subunit expressions of Ca_L_ channel in cerebral arteries between Diabetic + berberine and CON rats, which indicated that berberine treatment did not restore the α_1C_-subunit expressions of Ca_L_ channel in cerebral arteries of diabetic rat to the normal control level. When the control rats were treated with berberine (100 mg/kg/day), there was no significant difference in the α_1C_-subunit expressions of Ca_L_ channel in cerebral arteries between CON + berberine and CON rats. These results suggested that berberine treatment significantly reduced the augmented α_1C_-subunit expressions of Ca_L_ channel in cerebral arteries isolated from diabetic rats.

### Chronic administration of 100 mg/kg/day berberine not only markedly decreased the increased resting [Ca^2+^]_i_ level, but also suppressed the augmented Ca^2+^ releases from RyRs in cerebral VSMCs isolated from diabetic rats

**Fig. 6:**
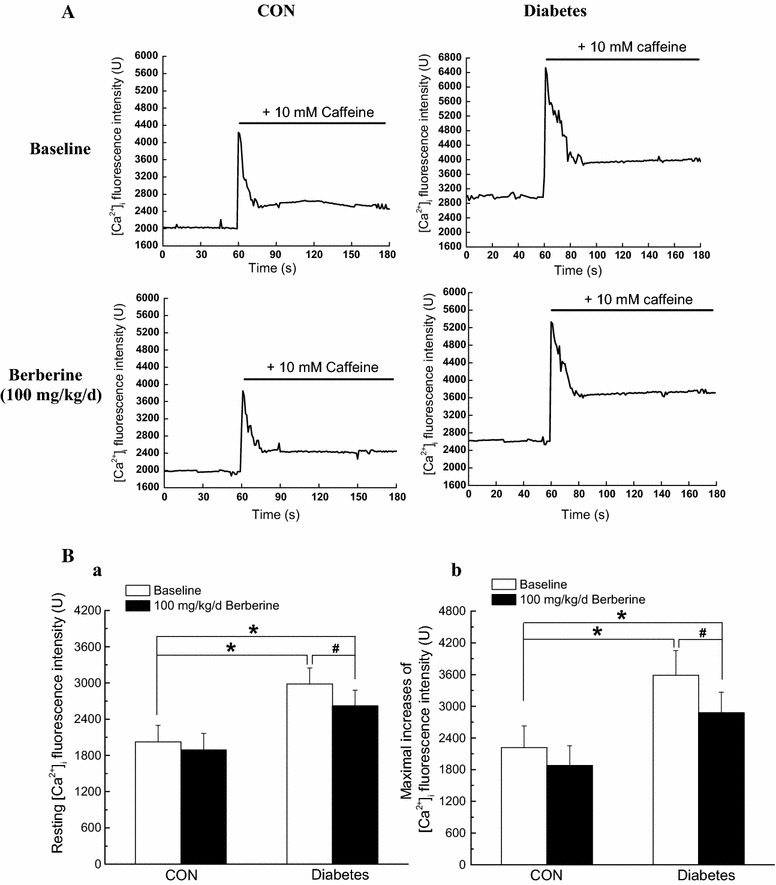
Comparison of resting level of Ca^2+^ fluorescence intensity and maximal increases of Ca^2+^ fluorescence intensity in cerebral VSMCs isolated from CON, CON +100 mg/kg/day berberine, Diabetic, and diabetic +100 mg/kg/day berberine rats. Representative *dot graph* of Fluo-3/AM fluorescence recorded showed the typical transient increases of intracellular Ca^2+^ fluorescence intensity evoked by 10 mM caffeine (**A**). Summarized data indicated the average changes of the intracellular Ca^2+^ fluorescence intensity before and during the application of caffeine (**B**). Chronic administration of 100 mg/kg/day berberine significantly not only decreased the resting [Ca^2+^]_i_ level, but also suppressed the Ca^2+^ releases from RyRs in cerebral VSMCs isolated from diabetic rats. *CON* control rats, *CON* *+* *berberine* control rats administrated with berberine (100 mg/kg/day), *Diabetic* diabetic rats, *diabetic* *+* *berberine* diabetic rats administrated with berberine (100 mg/kg/day). Values are expressed as mean ± SEM and at least n = 10 cells recorded in each group. *P < 0.05 vs. CON rats and^ #^ *P* < 0.05 vs. diabetic rats

As previously described [21], a high concentration of caffeine (10 mM) was used to activate RyRs and then evoked a transient peak increase of [Ca^2+^]_i_ which represents the Ca^2+^ release from the SR. Representative dot graph of Fluo-3/AM fluorescence recorded showed the typical transient increases of intracellular Ca^2+^ fluorescence intensity evoked by 10 mM caffeine (Fig. 6A). Summarized data indicated the average changes of the intracellular Ca^2+^ fluorescence intensity before and during the application of caffeine (Fig. 6B). We investigated the resting [Ca^2+^]_i_ level and the maximal caffeine-induced increases of [Ca^2+^]_i_ in cerebral VSMCs isolated from CON, CON + berberine, Diabetic, and Diabetic + berberine rats. As compared with that of CON rats, there is a significant higher resting [Ca^2+^]_i_ level in cerebral VSMCs isolated from diabetic rats, which is consistent with previous report that high glucose contributes to elevate the global [Ca^2+^]_i_ in VSMCs [4, 5]. The maximal increases of [Ca^2+^]_i_ were also significantly increased in cerebral VSMCs isolated from diabetic rats as compared with that of CON rats. In *Experiment I*, chronic administration of berberine (100 mg/kg/day) for 8 weeks not only significantly decreased the resting [Ca^2+^]_i_ level, but also markedly suppressed the maximal increases of [Ca^2+^]_i_ in cerebral VSMCs isolated from diabetic rats. However, there were also significant differences in the resting [Ca^2+^]_i_ level and the maximal increases of [Ca^2+^]_i_ in cerebral VSMCs between diabetic + berberine and CON rats, which indicated that berberine treatment did not restore the resting [Ca^2+^]_i_ level and the Ca^2+^ releases from RyRs in cerebral VSMCs of diabetic rats to the normal control level. When the control rats were treated with berberine (100 mg/kg/day), there was no significant difference in the resting [Ca^2+^]_i_ level and the Ca^2+^ releases from RyRs in cerebral VSMCs between CON + berberine and CON rats. These results indicated that berberine treatment not only markedly decreased the increased resting [Ca^2+^]_i_ level, but also suppressed the augmented Ca^2+^ releases from RyRs in cerebral VSMCs isolated from diabetic rats.

**Fig. 7:**
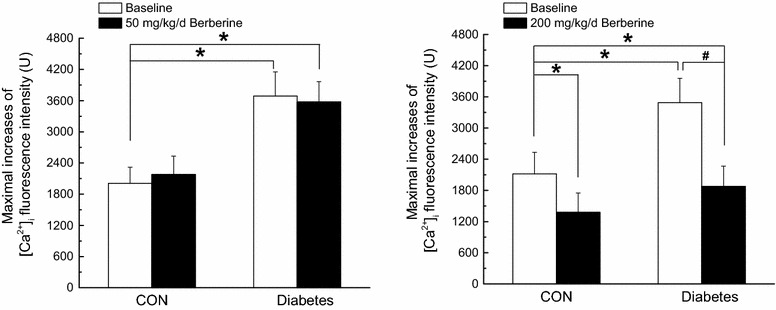
Comparison of maximal increases of Ca^2+^ fluorescence intensity in response to different dose of berberine (50 and 200 mg/kg/day) in cerebral VSMCs isolated from CON, CON + berberine, Diabetic, and diabetic + berberine rats. Chronic administration of 50 mg/kg/day berberine had no obvious effects on maximal increases of Ca^2+^ fluorescence intensity in CON and diabetic rats, respectively. In addition, chronic administration of 200 mg/kg/day berberine significantly inhibited the Ca^2+^ releases from RyRs in both CON and diabetic rats, respectively. *CON* control rats, *CON* *+* *berberine* control rats administrated with berberine, *Diabetic* diabetic rats, *diabetic* *+* *berberine* diabetic rats administrated with berberine. Values are expressed as mean ± SEM and at least n = 8 cells recorded in each group. *P < 0.05 vs. CON rats and^ #^ *P* < 0.05 vs. diabetic rats

In *Experiment II* and *Experiment III,* chronic treatment with 50 mg/kg/day berberine for 8 weeks had no obvious effects on maximal increases of Ca^2+^ fluorescence intensity in CON and diabetic rats, respectively. However, 200 mg/kg/day berberine for 8 weeks significantly inhibited maximal increases of Ca^2+^ fluorescence intensity in both CON and diabetic rats (Fig. 7).

### Acute application of berberine directly inhibited the hyperglycemia-induced Ca_L_ currents in cerebral VSMCs isolated from normal rats

To determinate whether the inhibition of Ca_L_ channel in diabetic rats is mainly due to a direct effect of berberine or only a secondary consequence of the lowing blood glucose in response to berberine treatment, we investigated the acute effects of berberine on the function of Ca_L_ channel in cerebral VSMCs isolated from normal control rats under high glucose condition. As shown in Fig. 5, extracellular application of the high glucose (d-glucose, 20 mM for 2–7 min, as described previously [5]) significantly evoked an increase of Ca_L_ channel currents at +10 mV test potential. Subsequently, acute extracellular application of 10 μM berberine (as described previously [23]) markedly reduced the amplitude of the inward currents in the presence of 20 mM d-glucose. These findings suggested that hyperglycemia increased Ca_L_ channel current and then the acute extracellular application of berberine could directly reduced the hyperglycemia-induced Ca_L_ currents in cerebral VSMCs of normal control rats.

### Acute application of berberine directly suppressed the hyperglycemia-induced Ca^2+^ releases from RyRs in cerebral VSMCs isolated from normal rats

To determinate whether the suppression of Ca^2+^ releases from RyRs diabetic rats is mainly due to a direct effect of berberine or only a secondary consequence of the lowing blood glucose in response to berberine treatment, we investigated the acute effects of berberine on the Ca^2+^ releases from RyRs in cerebral VSMCs isolated from normal control rats under high glucose condition. After the load of Fluo-3/AM, the isolated VSMCs were incubated with 20 mM d-glucose and the combination of 10 μM berberine and 20 mM d-glucose for 10 min at 37 °C, respectively. As shown in Fig. 6, incubation with 20 mM d-glucose significantly increased the resting [Ca^2+^]_i_ level (Fig. 6Ba) and maximal increases of [Ca^2+^]_i_ fluorescence intensity (Fig. 6Bb). However, incubation with combination of 10 μM berberine and 20 mM d-glucose markedly suppressed the resting [Ca^2+^]_i_ level (Fig. 6Ba) and maximal increases of [Ca^2+^]_i_ fluorescence intensity (Fig. 6Bb). These findings suggested that hyperglycemia increased the resting [Ca^2+^]_i_ and Ca^2+^ releases from RyRs and then the acute application of berberine could directly reduced the hyperglycemia-induced Ca^2+^ releases from RyRs in cerebral VSMCs of normal control rats.

## Discussion

There are two principal and novel findings in the present work. First, the berberine treatment could inhibit the increased cerebrovascular contractility independent of a functional endothelium in the rat model of streptozotocin-induced diabetes. Secondly, the berberine treatment not only inhibited the function of Ca_L_ channel, but also suppressed the Ca^2+^ releases from the RyRs of cerebral VSMCs in diabetic rats or when exposed to hyperglycemia condition. Our study indicated that berberine alleviated the cerebral arterial contractility in the rat model of streptozotocin-induced diabetes via regulating the intracellular Ca^2+^ handling of smooth muscle cells.

### Hyperglycemia impaired the intracellular Ca^2+^ handling in diabetic vascular dysfunction

Diabetic vascular complication has been demonstrated to be not only responsible for the increased risk of stroke and heart attack, but also contribute to the diabetic organ damage in diabetic patients [3]. Under diabetic conditions, multiple factors may intend to impair the function and structure of artery, including hyperglycemia, insulin resistance, hyperinsulinemia, excess free fatty acid (FFAs) release, advanced glycation end products (AGEs), lipemia, and obesity [1, 2]. However, hyperglycemia is still considered to be the most important feature in the development of diabetic vascular complication.

**Fig. 8:**
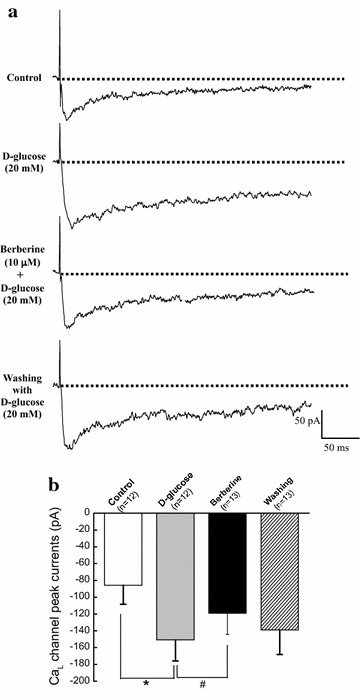
Acute application of berberine directly inhibited the hyperglycemia-induced Ca_L_ currents in cerebral VSMCs isolated from normal control rats. Representative traces of Ca_L_ channel current at +10 mV in cerebral VSMCs when exposed to 20 mM d-glucose and then subsequent acute application of 10 μM berberine in the presence of 20 mM d-glucose. At last, the cerebral VSMCs were washed with 20 mM d-glucose (**a**). Summarized data indicated the amplitudes of Ca_L_ channel current at +10 mV before and after application of 20 mM d-glucose, 10 μM berberine in the presence of 20 mM d-glucose, and then washing with 20 mM d-glucose (**b**). Values are mean ± SEM with the number of cells recorded in parentheses. *P < 0.05 vs. control condition and^ #^ *P* < 0.05 vs. high glucose condition

**Fig. 9:**
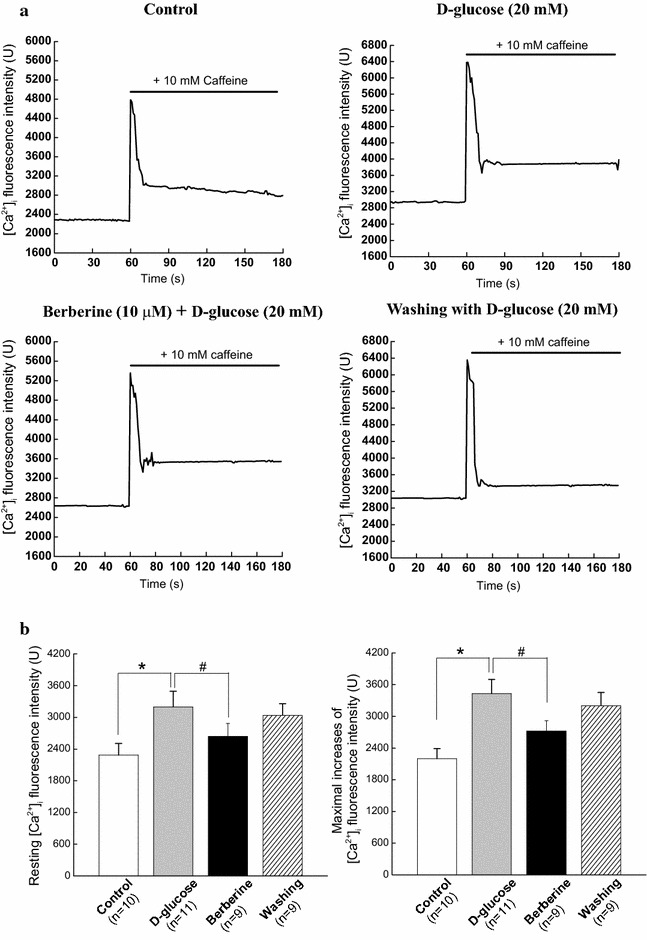
Acute application of berberine directly suppressed the hyperglycemia-induced Ca^2+^ releases from RyRs in cerebral VSMCs isolated from normal control rats. Typical transient increases of intracellular Ca^2+^ evoked by 10 mM caffeine were recorded when cerebral VSMCs exposed to the 20 mM d-glucose or the combination of 10 μM berberine and 20 mM d-glucose, respectively. At last, the cerebral VSMCs were washed with 20 mM d-glucose (**a**). Summarized data indicated the average changes of the resting intracellular Ca^2+^ and Ca^2+^ releases from RyRs before and after application of the 20 mM d-glucose and the combination of 10 μM berberine and 20 mM d-glucose, respectively (**b**). Values are mean ± SEM with the number of cells recorded in parentheses. *P < 0.05 vs. control condition and^ #^ *P* < 0.05 vs. high glucose condition

Ca_L_ channels in plasma membrane and RyRs in SR are important mediators to control arterial excitation–contraction coupling by handling intracellular Ca^2+^ in VSMCs. It is currently believed that when the membrane is depolarized, extracellular Ca^2+^ entries through Ca_L_ channels and then activates RyRs to produce the transient local Ca^2+^ release events, which is termed Ca^2+^-induced Ca^2+^ release (CICR). This Ca^2+^ event not only contribute to the overall rise in the concentration of intracellular Ca^2+^, but also in turn activate nearby Ca^2+^-activated K^+^ (K_Ca_) channels in plasma membrane, leading to membrane hyperpolarization, inhibition of Ca_L_ channels, and thereby favoring vasodilation by reducing the Ca^2+^ influx. In the present work, the rat model of STZ-induced diabetic rats showed a higher blood glucose level (Table 1) and a significant increase in the contractile function of cerebral artery (Fig. 1) as compared with that in CON. In addition, the function of Ca_L_ channel (Figs. 3, 5), the resting [Ca^2+^]_i_ level (Fig. 6), and the Ca^2+^ releases from RyRs (Fig. 6) significantly increased in cerebral VSMCs isolated from STZ-induced diabetic rats. Furthermore, we also observed that hyperglycemia directly increased the function of Ca_L_ channel (Fig. 8) and the Ca^2+^ releases from RyRs (Fig. 9) in cerebral VSMCs isolated from normal control rats. All these results are in agreement with previous reports that hyperglycemia induced diabetic vascular dysfunction by elevating global intracellular calcium ([Ca^2+^]_i_) [4, 5], up-regulating the function of Ca_L_ channel [5, 22], and altering the function of Ca^2+^ releases from the RyRs [6, 24]. It is also reported that cardiac dysfunction in diabetic cardiomyopathy is associated with a time-dependent decline in Ca^2+^ sparks of diabetic SD rat [25]. However, overexpression of silent information regulator 1 (SIRT1) reduces diabetes-exacerbated injury of myocardial ischemia and reperfusion and oxidative stress [26]. In addition, corin, a cardiac serine protease, also exerts cardioprotective action via activating pro-atrial natriuretic peptide pathway in diabetic cardiomyopathy [27].

### The berberine treatment alleviates the cerebrovascular contractility in diabetic rats by regulating the intracellular Ca^2+^ handling in VSMCs

Berberine [18, 5, 6-dihydro-9, 10-dimethoxybenzo (g)-1, 3-benzodioxolo (5, 6-a) quinolizinium] is an isoquinoline alkaloid, which is extracted from berberine-containing herbs, such as Goldenseal (Hydrastis canadensis), Oregon Grape (Berberis aquifolium), and Coptis chinensis (Chinese goldthread) [7]. It has been demonstrated that berberine exhibits a variety of pharmacological properties and has been extensively used for the immune enhancement, antibacterial and antiparasitic effects, and gastrointestinal infections [12]. Recently, berberine was found to have a long-term effect on lowering blood glucose, which could delay the diabetic complications in patients and various animal models [9]. The anti-hyperglycemic mechanisms of berberine are very complex, including the improving insulin secretion and sensitivity, activating the adenosine monophosphate-activated protein kinase (AMPK) pathway, modulating gut microbiota, reducing intestinal absorption of glucose, inhibiting gluconeogenesis and glucose transporter in liver, stimulating glycolysis in peripheral tissue cells [8, 12]. In addition, extensive studies indicated that berberine treatment also exerts an extra-benefit on the diabetic cardiovascular complications. Besides the anti-hyperglycemic and cholesterol-lowering activity, berberine also has the anti-inflammatory and anti-oxidant properties to protect hyperglycemia-induced endothelial injury [10, 12]. For example, berberine could improve the endothelium-dependent vasodilatation by activation of the AMPK pathway [14], increasing the phosphorylation of endothelial nitric oxide synthase (eNOS) [15, 28], down-regulating expression of NADPH oxidase [13], and inhibiting the formation of advanced glycation end products (AGEs) [29]. It has been demonstrated that berberine treatment attenuates palmitate-induced reduction in glucose uptake and consumption, in part, through reduction in cellular 1,2-diacyl-sn-glycerol (DAG) levels and accumulation of 1,2,3-triacyl-sn-glycerol (TAG) in H9c2 cells [30].

In the present work, chronic administration of 100 mg/kg/day berberine not only significantly reduced glucose levels in diabetic rats (Table 1), but also markedly inhibited the augmented contractile function of cerebral artery in diabetic rats (Fig. 1). In addition, chronic administration of 100 mg/kg/day berberine not only markedly inhibited the increased Ca_L_ channel current densities (Fig. 3), but also reduced the augmented α_1C_-subunit expressions of Ca_L_ channel (Fig. 5) in cerebral VSMCs isolated from diabetic rats. Furthermore, 100 mg/kg/day chronic administration of berberine not only markedly decreased the increased resting [Ca^2+^]_i_ level, but also suppressed the augmented Ca^2+^ releases from RyRs (Fig. 6) in cerebral VSMCs isolated from diabetic rats. Correspondingly, acute application of berberine could directly inhibit the hyperglycemia-induced Ca_L_ currents (Fig. 8) and suppress the hyperglycemia-induced Ca^2+^ releases from RyRs (Fig. 9) in cerebral VSMCs isolated from normal control rats. Our results suggested 100 mg/kg/day berberine treatment could alleviate the cerebral arterial contractility by regulating the intracellular Ca^2+^ handling of VSMCs in diabetic rats or when exposed to hyperglycemia condition.

From our results, chronic administration of 50 mg/kg/day berberine had no obvious effects on contractile responsiveness (Fig. 2), Ca_L_ peak current density (Fig. 4), and maximal increases of Ca^2+^ fluorescence intensity (Fig. 7) in CON and Diabetic rats, respectively. However, chronic administration of 200 mg/kg/day berberine significantly inhibited the contractile responsiveness (Fig. 2), Ca_L_ peak current density (Fig. 4), and maximal increases of Ca^2+^ fluorescence intensity (Fig. 7) in both CON and diabetic rats, respectively. We concluded that the dose of berberine is very important for treatment and 100 mg/kg/day berberine could produce significant effects on DM rats rather than CON rats in terms of contractile function, Ca_L_ channel and [Ca^2+^]_i_.

### Practical implications of the present study

It has been reported that several therapeutic strategies are administered to ameliorate the deteriorated vascular function in diabetes, such as increasing the endothelium-dependent NO production, reducing the oxidative stress damage, inhibiting the inflammatory responses [7, 8]. Our study may provide a novel therapeutic approach to treat vascular dysfunction in diabetic patients by regulating the intracellular Ca^2+^ handling with their related proteins in VSMCs. In addition, the safety and efficacy of berberine have been generally accepted as a kind of traditional Chinese medicine. It is believed that berberine performs a good clinical practice and our study provided new evidence that application of berberine is a novel potential application in the treatment of diabetic vascular dysfunction.

### Study limitations

First, there are some disparities between our work and other previous reports. The present work showed that the function of Ca_L_ channel significantly increased in the rat model of STZ-induced diabetic rats (Figs. 3, 5) or when exposed to hyperglycemia condition (Fig. 8), which are in consistence with previous reports [5, 22]. However, there is also a report that Ca_L_ channel currents significantly reduced in STZ-induced diabetic rats [3]. In addition, we observed that Ca^2+^ releases from RyRs significantly increased in the diabetic rats (Fig. 6) or when exposed to hyperglycemia condition (Fig. 9), which are in agreement with previous report that there was a dramatic increase in ryanodine receptor (RyR) levels of VSMCs from diabetic animals [6]. However, reduced expression of RyR has also been reported in several diabetic models [3]. These discrepancies may be related to different species or rat strain, the method of diabetic induction, the vascular bed studied or the severity of diabetes [3, 31]. Second, RyRs and inositol 1,4,5-trisphosphate receptors (IP3Rs) are the two mainly important families of Ca^2+^ release channels in the SR. Activation of IP_3_Rs could also evoke the localized Ca^2+^ transients and contribute to the global elevation of intracellular Ca^2+^. Thus, it is necessary to study the role of IP_3_Rs in the berberine treatment for the diabetic vascular dysfunction. Third, we observed that berberine inhibited the function of Ca_L_ channel in the present work. However, the present work did not study the underlying mechanism. It is believed that there are three subclasses of calcium channel blockers which are wildly used to block Ca_L_ channel by binding to the poreforming α1C subunit, such as dihydropyridine, phenylalkylamine, and benzothiazepine. Whether berberine inhibited the function of Ca_L_ channel by directly binding to poreforming α1C subunit or other indirect pathway needs the further investigation.

## Conclusion

The present work showed that berberine treatment could alleviate the cerebral arterial contractility independent of a functional endothelium in the rat model of streptozotocin-induced diabetes. In addition, the berberine treatment not only directly inhibited the function of Ca_L_ channels, but also suppressed the function of Ca^2+^ releases from the RyRs of cerebral VSMCs in diabetic rats or when exposed to hyperglycemia condition. Taken together, our study indicated that berberine alleviated the cerebral arterial contractility in the rat model of streptozotocin-induced diabetes via regulating the intracellular Ca^2+^ handling of smooth muscle cells.

## Abbreviations

DM: diabetes mellitus
[Ca^2+^]_i_: concentration of intracellular Ca^2+^
VSMCs: vascular smooth muscle cells
Ca_L_: long-lasting voltage-dependent Ca^2+^ (L-type Ca^2+^) channel
RyRs: ryanodine receptors
STZ: streptozotocin
5-HT: 5-hydroxytryptamine
CICR: Ca^2+^-induced Ca^2+^ release
K_Ca_: Ca^2+^-activated K^+^ channel
AMPK: adenosine monophosphate-activated protein kinase
eNOS: endothelial nitric oxide synthase
AGEs: advanced glycation end products
